# Aqueous and Ethanolic Plant Extracts as Bio-Insecticides—Establishing a Bridge between Raw Scientific Data and Practical Reality

**DOI:** 10.3390/plants10050920

**Published:** 2021-05-04

**Authors:** Wilson R. Tavares, Maria do Carmo Barreto, Ana M. L. Seca

**Affiliations:** 1 cE3c—Centre for Ecology, Evolution and Environmental Changes/Azorean Biodiversity Group & Faculty of Sciences and Technology, University of Azores, Rua Mãe de Deus, 9501-321 Ponta Delgada, Portugal; wilson.r.tavares@uac.pt; 2 LAQV-REQUIMTE, Department of Chemistry, University of Aveiro, 3810-193 Aveiro, Portugal

**Keywords:** bio-insecticides, aqueous extracts, ethanolic extracts, plant extracts, crop pest management, *Azadirachta indica*, *Capsicum annuum*, *Nicotiana tabacum*, *Tagetes erecta*

## Abstract

Global demand for food production is causing pressure to produce faster and bigger crop yields, leading to a rampant use of synthetical pesticides. To combat the nefarious consequences of its uses, a search for effective alternatives began in the last decades and is currently ongoing. Nature is seen as the main source of answers to crop protection problems, supported by several examples of plants/extracts used for this purpose in traditional agriculture. The literature reviewed allowed the identification of 95 plants whose extracts exhibit insecticide activity and can be used as bio-pesticides contributing to sustainable agriculture. The option for ethanol and/or water extracts is more environmentally friendly and resorts to easily accessible solvents, which can be reproduced by farmers themselves. This enables a bridge to be established between raw scientific data and a more practical reality. *Azadirachta indica*, *Capsicum annuum*, *Nicotiana tabacum* and *Tagetes erecta* are the most researched plants and have the potential to be viable options in the pest management approach. *Azadirachta indica* showed the most promising results and *Brevicoryne brassicae* was the most targeted pest species, being tested against the aqueous and/or ethanolic extracts of 23 different plants. Maceration using dried material (usually leaves) is the extraction method preferred by the majority of authors.

## 1. Introduction

Agriculture was fundamental in the development of human society, enabling a gradual shift from a hunter-gatherer nomadic lifestyle to a more settled way of life, allowing primordial civilizations to flourish [1]. Since then, its role in the success of humans has only grown, and currently its importance is at its peak due to the globally rising demand for food production [2]. In recent years, there has been an unsustainable intensification of agriculture, causing an increase in the use of chemicals to allow bigger, faster and pest-free crop productions [3].

The emergence of insect pests in crops represents a serious problem, since they decrease the yield of production leading to considerable losses of time and money invested [4]. Synthetic insecticides are commonly chosen to tackle that problem, since they offer a solution that is often quick and effective, acting almost instantly and not requiring much labour-intensive work to be applied [5,6]. Unfortunately, the continued use of these chemicals can lead to various environmental problems [7,8], with harmful consequences for various living beings [9,10,11] and also affecting human health [12,13,14,15]. Additionally, the intensive use of synthetic pesticides in the last century has caused an increase in resistance by crop pests [16], with reports of insecticide resistance going as far back as 1897, when John B. Smith reported the observation of resistance to kerosene of the San Jose scale (*Quadraspidiotus perniciosus*) [17].

To avoid the negative points inherent to the use of synthetic insecticides, it is necessary to find more efficient alternatives with less environmental impact that can control the pest populations [18]. Furlan and colleagues [19] reviewed the various alternatives to systemic insecticides in agriculture. From landscape manipulation to changes in the established farming practices, or using specific organisms to combat the pests or traps to catch them, the pest management tactics are diverse [19]. One of those tactics is the use of naturally derived insecticides, such as plant extracts, that are more environmentally friendly and sustainable than the commercial synthetic insecticides widely used in agriculture [20]. According to their production method, botanical insecticides can be grouped in two major categories: i.e., commercial botanical insecticides and farm products. The first group is composed of products developed by companies with a commercial aim, basing their formulations on active substances obtained from selected plant species with reported insecticide properties. The second group consists of products produced by rural farmers according to traditional recipes and/or popular knowledge of pesticidal effects of some plants in the area of their local use passed on for generations [21].

As an evolutionary response to the environment, plants can modulate their behaviour in order to succeed in nature [22], developing specialized morphological structures and synthesizing several products to try to ensure their survival against insects and other predators [23,24]. Secondary metabolites are produced, with specific properties against different insects, such as antinutritional, repellent, and/or toxic effects [25]. Humans have always observed these effects and started to experiment with different plants to take advantage of these insecticide properties to protect crops. For example, the ancient Greeks and Romans believed that if seeds were sowed just before a new moon and after being mixed with crushed cypress (*Cupressus* spp.) leaves, they would be protected from maggots [26]. One of India’s oldest documents describes the use of several plants to protect crops, such as a powdered mixture of the roots of five plants (i.e., *Aegle marmelos* (L.) Corrêa, *Clerodendrum phlomidis* L.f., *Gmelina arborea* Roxb., *Stereospermum chelonoides* (L.f.) DC. (syn. *Stereospermum suaveolens* (Roxb.) DC.) and *Oroxylum indicum* (L.) Kurz) which was used to restore plant health [27].

The use of plants to protect crops against insect pests is not just a practice of ancient civilizations. This practice continues to be used by farmers around the world, mainly in areas where access to synthetic pesticides is more difficult and in organic farming, using plants in the form of extracts, company plants or just the harvested plant [28,29,30,31]. Some examples of this reality are presented below.

In Ghana, chilli peppers (*Capsicum annuum* L.) and orange (*Citrus sinensis* (L.) Osbeck) peel are well-known for their protection effect of stored crops against insect pests, as well as *Azadirachta indica* A.Juss., *Cymbopogon schoenanthus* (L.) Spreng., *Securidaca longipedunculata* Fresen. and *Senna sophera* (L.) Roxb. (syn. *Cassia sophera* L.) [28]. Most of the plants are used individually or in a mixture with other pesticidal plants, after being pounded and turned into a powder to mix with the crop before storage, although other methods of application are described [28]. Local farmers in Kenya use aqueous concoctions of *Azadirachta indica* and *Capsicum annuum* for insect pest control [32]. In addition to the aqueous extracts, ashes from cow dung mixed with the pesticidal plants are also used to protect stored crops [32]. According to the rural farmers in Malawi and Zambia, the cultural practices against insect pests using plants mainly involve the use of locally available plants known for their insecticide and repellent properties, such as *Azadirachta indica*, *Bobgunnia madagascariensis* (Desv.) J.H.Kirkbr. & Wiersema, *Euphorbia tirucalli* L., *Nicotiana tabacum* L., *Tithonia diversifolia* (Hemsl.) A.Gray, *Toona ciliata* M.Roem. and *Tephrosia vogelii* Hook.f., being *Tephrosia vogelii* the most widely used plant in both regions [33,34]. These plants are particularly effective against bollworms and red spider mites affecting tomato crops and against aphids, diamond back moths and webworms affecting cruciferous vegetables crops [34]. In addition, powdered *Bobgunnia madagascariensis* is popular in Malawi for its molluscicide properties [35].

In Sri Lanka, *Croton laccifer* L. branches are detached from the plant and beaten to damage their leaves, originating a strong odour that repels rice bugs present in rice crops. Another technique used to repel these bugs is to burn chipped wood of *Cerbera manghas* L. near the rice fields [36]. Some communities in India store their crops in cylindrical basket-like structures made with *Borassus flabellifer* L. leaves tightly woven to prevent the entry of insects. In addition, leaves of local plants known to have insect deterrent action are used as inner lining of the basket, i.e., *Azadirachta indica*, *Psidium guajava* L., *Vitex negundo* L. and *Pongamia pinnata* (L.) Pierre [37]. Indian communities in the south of the country report other pest control techniques, such as the placement of leaves of *Azadirachta indica*, *Coriandrum sativum* L., *Leucas aspera* (Willd.) Link or *Pongamia pinnata* as layers between the crop sacks stacked one above the other in storehouses [38]. According to the locals, the strong odour of the leaves is effective in keeping away pests such as *Cryptolestes minutus*, *Ephestia cautella*, *Latheticus oryzae*, *Oryzaephilus surinamensis*, *Rhyzopertha dominica* and *Tribolium castaneum*. *Acorus calamus* L., *Azadirachta indica* and lime (*Citrus* spp.) powders can also be used for the same purpose [38]. Another example is the application of *Areca catechu* L. fruits and leaves suspended from wooden beams in small storages of rice to repel insect pests in rural farming communities of the Philippines [39]. To control the damage of cotton plantations (*Gossypium* spp.) by the bollworm (*Helicoverpa armigera*), farmers in Benin use mixtures of *Hyptis suaveolens* (L.) Poit., *Khaya senegalensis* (Desv.) A.Juss. and *Azadirachta indica* to spray on the cotton plants [40].

In Cameroon, *Cannabis sativa* L. is widely used by farmers of cacao plantations (*Theobroma cacao* L.) to control cacao pests, particularly capsids insect populations, being planted side-by-side with the cacao plant to act as a deterrent for the pests [41]. Furthermore, the *Cannabis sativa* aqueous extract is obtained from fresh or dried pounded leaves and is applied alone or in mixture with other local insecticidal plants extracts (i.e*., Ceiba pentandra* (L.) Gaertn., *Erythrophleum ivorense* A.Chev., *Guibourtia tessmannii* (Harms) J.Leonard, *Nicotiana tabacum* and *Pachyelasma tessmannii* (Harms) Harms). According to the farmers, the number of species used influences the effectiveness of the extracts, the more diversified the extract mixture, the better [41]. Similarly, in Uganda, the use of mixtures of various plant parts (bark, flowers, leaves, roots, seeds and stems) from different plant species (*Azadirachta indica*, *Cannabis sativa*, *Capsicum annuum* L. (syn. *Capsicum frutescens* L.), *Cupressus lusitanica* Mill., *Moringa oleifera* Lam., *Musa* spp., *Nicotiana tabacum*, *Tagetes erecta* L., *Tagetes minuta* L. and *Tephrosia vogelii*) is common for field and stored crop protections [42]. In this case, the plants alone or mixtures of them are burnt to obtain plant ash that is then applied on the crops to control insect pests such as stem borer (*Busseola fusca*), banana weevil (*Cosmopolites sordidus*), bean fly (*Ophiomyia phaseoli*) grain moth (*Sitotroga cerealella*), pod borers (*Maruca vitrata* and *Nezara viridula*) and aphids (*Aphis craccivora*, *Aphis fabae* and *Rhopalosiphum maidis*) [42].

The traditional uses of plants as insecticides addressed above show that several species are used in different ways, depending on the location of the report, with some plants deeply rooted in popular knowledge being mentioned regularly, e.g., *Azadirachta indica* and *Nicotiana tabacum*. These reports do not usually allow for exact conclusions to be drawn regarding the effectiveness of the plants mentioned, since only the testimony of rural farmers is considered, lacking, in many cases, scientific evidence to support their claims. However, these information demonstrate the value of popular knowledge in providing scientists with good starting points in their search for promising sources of new bioactive natural compounds with insecticide properties [43,44].

In addition, pure compounds with insecticidal action have been isolated from several plants, such as Azadirachtin (isolated from *Azadirachta indica*) and pyrethrins (isolated from *Tanacetum cinerariifolium* (Trevir.) Sch.Bip.), which are two cases of successful natural insecticides, being the base of the majority of current commercial botanical insecticides [45,46,47]. However, this kind of insecticides tends to be difficult to obtain by most smallholder farmers since they are relatively expensive, making crops economically unsustainable [48].

This review was elaborated to provide reliable scientific information regarding plants with insecticide properties to anyone who wants to try a more natural approach to pest management. To promote sustainable practices, this work contemplates only studies performed with easily accessible and environmentally friendly solvents, such as ethanol and water, leaving out the literature on insecticide activities exhibited by other organic extracts, essential oils and pure compounds. The research for this review was made crossing the terms bio-insecticide, ethanol plant extracts, water plant extracts, insecticide activity and crop pests in the databases Web of Science, PubMed and Scopus, and only the published works involving plant species with an accepted binominal Latin name on the “The Plant List” database were considered [49].

## 2. Plant Extracts as Bio-Insecticides

To be a viable alternative to synthetic insecticides, bio-insecticides must be affordable (based on plant materials readily available and cheap) and be simple to prepare (not requiring complex equipment and solvents which are either toxic or difficult to buy) [50]. Furthermore, the resultant bio-insecticide should have low phytotoxicity, causing no negative effects on crop yields, being also benign to the natural enemies of the targeted pest, and avoiding resurgent and new pests [51,52]. Thus, in this next section, aqueous and ethanolic plant extracts with reported insecticidal properties against common crop pests, and which could be considered as potential effective bio-insecticides, will be discussed.

In recent years, various works have been carried out to ascertain whether some plant extracts have the insecticide potential to become a reliable choice over synthetic insecticides, with some of them showing very interesting results [47,53]. The bibliographic research carried out revealed some works whose experimental design, activity level and/or conclusions deserve a more detailed discussion, being presented below.

### 2.1. Aqueous Extracts

To provide farmers with quick and cheap access to crop pest control solutions, Mkenda et al. [54] analysed the insecticide potential of four abundant plant species found in Tanzania. Aqueous leaf extracts of *Tephrosia vogelii*, *Tithonia diversifolia*, *Vernonia amygdalina* Delile and *Lippia javanica* (Burm.f.) Spreng. provided effective control against common pests of the bean plant *Phaseolus vulgaris* L., i.e., aphids (*Aphis fabae*), flower beetles (*Epicauta albovittata* and *Epicauta limbatipennis*) and bean foliage beetles (*Ootheca mutabilis* and *Ootheca bennigseni*). *Vernonia amygdalina* extract provided control of flower beetles comparable to the synthetic insecticide Karate^®^. In fact, average insect abundance using *Vernonia amygdalina* extract (1 and 10% *w*/*v*) was 0.63, using Karate^®^ (2.5 mg/mL) it was 0.37, while in the untreated plants it was 2.45. *Lippia javanica* and *Vernonia amygdalina* were generally more effective in reducing pest insect incidence, abundance and damage than *Tephrosia vogelii* and *Tithonia* *diversifolia*. However, treatments with *Tephrosia vogelii* and *Tithonia diversifolia* produced significantly higher crop yields than all other treatments, which the authors attributed to the lowest impact of these two treatments on the numbers of auxiliary agents, such as lady beetles and spiders [54]. The impact of a bio-pesticide on auxiliary control agents is a valuable and interesting variable that should be taken into account in works that evaluate the insecticide potential of plant extracts, but which unfortunately is often overlooked by researchers.

In a study involving the aphid *Brevicoryne brassicae* [55], nine aqueous extracts were tested for their repellent and insecticide properties. Two different extraction techniques (cold extraction of dry material and infusion of fresh material) and different parts of the plants (flowers, fruits and leaves) were used to obtain the plant extracts that were further sprayed at different concentrations (1, 2.5, 5 and 10% *w*/*v*) over the studied insects. The most effective extracts as repellents were the ones from the fresh fruits of *Solanum guaraniticum* A. St.-Hil. (syn. *Solanum fastigiatum* var. *acicularium* Dunal) and *Solanum pseudocapsicum* L. (syn. *Solanum diflorum* Vell.) (both at 2.5 and 5% concentrations). Leaf extracts of *Solanum guaraniticum* and *Solanum bonariense* L. (syn. *Solanum fastigiatum* var. *fastigiatum*) (10% concentration) demonstrated to be the most effective treatments regarding the insecticide capacity, affecting the reproduction and survival of *Brevicoryne brassicae*.

Rando et al. [56] assessed the insecticide properties of water extracts (at 10% *w*/*v*) of *Coriandrum sativum*, *Equisetum hyemale* L., *Nicotiana tabacum* and *Ocimum gratissimum* L., sprayed against *Brevicoryne brassicae* and *Myzus persicae*. The results showed that only *Coriandrum sativum* and *Nicotiana tabacum* extract were effective after 72 h of treatment against both pests, presenting mortality rates similar to the organophosphate insecticide acephate used as control. *Equisetum hyemale* and *Ocimum gratissimum* also presented insecticide properties but only against *Brevicoryne brassicae*.

*Nicotiana megalosiphon* Van Heurck & Müll.Arg. was studied to assess the insecticidal properties of its aqueous extract against the common pests of cabbage (*Brassica oleracea* L.), such as the cabbage aphid (*Brevicoryne brassicae*), the green peach aphid (*Myzus persicae*) and the diamondback moth (*Plutella xylostella*) [57]. The extracts at 1%, 5% and 10% *w*/*v* (grams of plant per 100 mL of tap water) were able to control the population numbers of all pest species, presenting the highest effect against *Plutella xylostella* (mortality of 25 ± 0.03%, 90 ± 0.04% and 100 ± 0.00%, respectively), 24 h after treatment, being better than the reference insecticide tau-fluvalinate at 7.5 g/L diluted as recommended by the manufacturer to 9.5 mL/L, that just presented a mortality rate of 22 ± 0.05%.

*Aphis gossypii* was the target in the work of Santos et al. [58], where powdered *Azadirachta indica* seeds were added to distilled water at different concentrations (23.8, 122.0, 410.0 and 1,410.0 mg/100 mL). The results showed that the aqueous extracts could reduce the survival period of *Aphis gossypii* in a dose-dependent manner, with the higher concentration reducing the life expectancy of the insects from 17.4 to 2.5 days. Other works have also proved the great insecticide effect of *Azadirachta indica* extracts. Hernández-Castro et al. [59], showed the efficiency of aqueous extract 10% *w*/*v* (10 g of plant material per 100 mL of water) of unpeeled *Azadirachta indica* seed in repelling the aphid *Aphis nerii* from feeding on papaya plants (*Carica papaya* L.) sprayed with the plant extract. In addition, the aphid mortality after 24 h at the extract-sprayed plants was higher by 27% when compared to the control (only water-sprayed plants). Another work [60] also demonstrated the insecticidal properties of *Azadirachta indica* aqueous extract, being effective in lowering the numbers of *Aphis gossypii* after a 72 h period. In Hossain and Haque’s work [61], the water extract of *Azadirachta indica* seeds (10% v/w) proved to be effective in protecting chickpea (*Cicer arietinum* L.) seeds against the pulse beetle *Callosobruchus chinensis*, causing, after a seven days treatment, a significant decrease in the number of laid eggs per 100 seeds (22 ± 1.32), in the number of adult insects (41 ± 1.60) and in the infestation percentage (14.61%) when compared with the control (94.33 ± 1.97 eggs; 190 ± 2.28 adult insects; infestation percentage = 63.57%). The same study also showed that other 10 plant water extracts were effective in decreasing these parameters, but none as good as the *Azadirachta indica* extract.

In a recent study [62], ten aqueous plant extracts (10% *w*/*v*, i.e., 100 g of plant powder per litre of water) were evaluated regarding their insecticide potential against *Spodoptera frugiperda* larvae. The plants extracts were from *Azadirachta indica*, *Aloe vera* (L.) Burm.f., *Cymbopogon citratus* (DC.) Stapf, *Lantana camara* L., *Lippia javanica*, *Nicotiana tabacum*, *Ocimum basilicum* L., *Trichilia emetica* Vahl, *Tephrosia vogelii* and *Vernonia amygdalina*. The most effective extracts were *Azadirachta indica*, *Cymbopogon citratus*, *Lippia javanica*, *Nicotiana tabacum* and *Ocimum basilicum*, which caused at least 50% mortality. Despite the interesting outcomes, the authors presented the results as a graphic which does not allow for an accurate reading of numerical values, in addition to not using a reference insecticide as positive control to allow for comparison of results.

The corn earworm (*Helicoverpa zea*) and the fall armyworm (*Spodoptera frugiperda*) were used to test the insecticidal potential of *Peumus boldus* Molina aqueous extract at concentrations ranging from 0.25% to 8.0% v/v [63]. After 7 days of diet, the 8.0% concentration extract proved to be the most effective against the pests, causing a mortality of 30.0 ± 7.2% against *Helicoverpa zea* while *Spodoptera frugiperda* exhibited greater sensitivity to the extract with a mortality of 75.0 ± 6.5%.

The fumigant toxicity of *Allium sativum* L. aqueous extract (*w*/*v* at 1:1 ratio) against adults and larvae of *Tribolium castaneum* was assessed [64]. The results determined that the LC_50_ values for adults at 24 h and 48 h after exposure were of 127.90 µL/L air and 90.8 µL/L air and for larvae they were, respectively, 267.37 µL/L air and 145.8 µL/L air. Furthermore, repellent activity was also evaluated regarding the adults of *Tribolium castaneum*. A concentration of 2.13 µL/cm^2^ provided a repellence of 95 ± 7.07% two hours after exposure. Despite the interesting results, the authors chose to present data in an unconventional way. Conversions for more common units (e.g., µg/mL) should have been made to facilitate the reader to make conclusions and be able to compare the results obtained with other similar works.

Aqueous extracts of *Saponaria officinalis* L. roots (0.2 to 1.9% *w*/*v*) were evaluated regarding their acaricidal effect towards *Tetranychus urticae* and the results showed that they affected the development stages of the pest in a dose-dependent manner [65]. Adults revealed the lowest sensitivity (LC_50_ = 1.18% *w*/*v*), while eggs were the most sensitive (LC_50_ = 0.31% *w*/*v*). Furthermore, oviposition was also affected by the different extract concentrations, with a LC_50_ value established at 0.91% *w*/*v* concentration.

Amoabeng and colleagues [66] presented a study in which the insecticide potential of nine water-detergent extracts (*Ageratum conyzoides* (L.) L., *Capsicum annuum* (syn. *Capsicum frutescens*), *Chromolaena odorata* (L.) R.M.King & H.Rob., *Jatropha curcas* L., *Nicotiana tabacum*, *Ocimum gratissimum*, *Ricinus communis* L., *Senna sophera* (syn. *Cassia sophera*) and *Synedrella nodiflora* (L.) Gaertn. was assessed against both cabbage (*Brassica oleracea*) pests, the cabbage aphid (*Brevicoryne brassicae*) and the diamondback moth (*Plutella xylostella*). All plant extracts (3 g of plant per 100 mL of tap water resulting in 3% *w*/*v* final concentration with 0.1% Sunligth^®^ detergent) demonstrated to be effective in reducing the insect number of both pests in field cage and open field experiments, presenting results similar to the synthetic insecticide Attack^®^ (emamectin benzoate) used as control at 1.5 mL/L. In some cases, the plant extracts matched the effectiveness of the control insecticide, such as *Ageratum conyzoides* and *Chromolaena odorata* against *Plutella xylostella* (100% number reduction) and *Senna sophera*, *Jatropha curcas*, *Nicotiana tabacum*, *Ricinus communis* and *Synedrella nodiflora* against *Brevicoryne brassicae* (infestation score = 0). Furthermore, it should be mentioned that the plant extracts were much less toxic than the synthetic insecticide to the natural enemies of *Brevicoryne brassicae* and *Plutella xylostella* present in the study site, i.e., hoverflies, ladybirds and spiders, causing a smaller impact in the trophic chain.

*Brevicoryne brassicae* populations were also successfully controlled in an open field study [67] using a water-soap plant extract. An aqueous solution (5% concentration *w*/*v*) of powdered *Melia azedarach* L. seeds was prepared with 0.1% *w*/*v* of soap powder to be sprayed across selected cabbage crop plots. Throughout a six-week treatment period a proportionately increased overall percentage reduction in *Brevicoryne brassicae* populations was verified, from 19.06% in the first week to 86.5% in the last week of the study. Although populations of *Brevicoryne brassicae*’s natural enemy *Coccinella septempunctata* (i.e., ladybirds) were also reduced, these differences were not statistically significant (*p* > 0.05). Thus, the authors claim that *Melia azedarach* aqueous extract showed effective aphicide activity, while being safe to natural enemies of *Brevicoryne brassicae.*

Kestenholz and colleagues [68] investigated, in lab and in the field, the possible insecticide action of *Senna sophera* (syn. *Cassia sophera*) cold- and hot-water leaf extracts (12.5% *w*/*v*), protecting stored *Vigna unguiculata* (L.) Walp. against *Callosobruchus maculatus* and *Sitophilus oryzae* infestations. It was demonstrated that the *Senna sophera* hot-water extract was more effective in reducing the numbers of *Callosobruchus maculatus* than the cold-water extract. Regarding *Sitophilus oryzae*, it appears that the insect mortality was unaffected by the *Senna sophera* extracts since the number of insects was higher in the jars with the extract treated grains than in the control jar with untreated grains.

A field study [69] ascertained if the aqueous extracts of leaves and seeds from three plants (i.e*., Allium sativum*, *Swertia chirata* Buch.-Ham. ex Wall. and *Swietenia mahagoni* (L.) Jacq.) could grant insecticide protection to a cucumber (*Cucumis sativus* L.) field crop. All three extracts (100 g of plant material per litre of distilled water) were effective in protecting *Cucumis sativus* plants against different pests over a 2-month period, with *Swertia chirata* showing the best results with an average of 1.67 ± 0.18 leaves attacked, while the control (untreated plants) presented an average of 4.66 ± 0.33 leaves attacked.

Another field study [70] assessed the insecticide effects of a seven-day treatment of aqueous extracts at 5% *w*/*v* (50 mg of plant per litre of water) of rhizomes of *Zingiber officinale* Roscoe and leaves of *Anthemis cotula* L., *Artemisia annua* L., *Datura stramonium* L. and *Juglans regia* L., against *Mythimna separata* present on oats (*Avena sativa* L.) crops. After the treatment period, all studied plants reported mortality over 65% against the pest, with *Artemisia annua* being the most active, presenting a mortality rate of 84.59%, almost as good as the insecticide of reference Dichlorvos 76 EC (at concentration of 0.076%) that presented a mortality rate of 89.34%.

In another field study [71], the effectiveness of *Allium sativum* (50 mg of plant material per millilitre of distilled water) and *Capsicum annuum* (syn. *Capsicum frutescens*) aqueous extracts (70 mg of plant material per millilitre of distilled water) in the management of the major pests (*Brevicoryne brassicae*, *Hellula undalis*, *Plutella xylostella* and *Trichoplusia ni*) of cabbage (*Brassica oleracea*) was assessed. After a treatment of four weeks, the results show that both extracts were effective in lowering the population number of all pests, with *Brevicoryne brassicae* being the most affected by the *Allium sativum* aqueous extract (reduction of 42.05%) and *Hellula undalis* being the most affected by the *Capsicum annuum* aqueous extract (reduction of 55.94%). For term of comparison, the control insecticide Attack^®^ (emamectin benzoate at 2.5 mL/L) was also effective against all pest species, causing a reduction of 70.83% against *Brevicoryne brassicae* and of 60.06% against *Hellula undalis*.

In a field study [72], the number of cacao mirids (*Sahlbergella singularis*) were controlled using aqueous extracts obtained from the seeds of *Azadirachta indica* and *Cascabela thevetia* (L.) Lippold (syn. *Thevetia peruviana* (Pers.) K.Schum.), being as effective in reducing the insects in the field as the positive control Actara^®^25 WG (a benchmark insecticide). Over a period of 5 months both extracts, prepared using 14.7 g of plant per litre of water were applied over the cacao trees at 15 days intervals and the commercial insecticide was applied, as recommended, at concentration of 0.26 g/L once a month. Water, the negative control, was also applied once a month. The results show a decrease in the average number of mirids on each tree over the study period, which was similar for the two extracts (*Azadirachta indica*: from 5.67 ± 0.99 to 1.98 ± 0.14; *Cascabela thevetia*: from 5.33 ± 0.33 to 1.21 ± 0.11) and for the reference insecticide (from 4.83 ± 0.70 to 1.67 ± 0.13). For term of comparison, the results of the negative control increased from 6.33 ± 1.14 to 8.95 ± 1.18. To achieve comparable effectiveness, the application of the aqueous extracts needs to be twice as frequent as the application of the insecticide, but the authors suggest the adoption of their protocol by farmers for pest control in cacao plantations since it provides similar results while being more environmentally friendly, less risky for human health and cheaper [72]. Although this study is interesting, the authors do not provide enough information to convert the extract preparation data into a dry extract concentration, making it impossible to compare with other studies in which the applied concentration is the extract mass by volume and not plant mass by volume.

Fite et al. [73] carried out a very complete field study. They found that the administration of the aqueous extracts of *Azadirachta indica* or *Millettia ferruginea* (Hochst.) Baker, individually (5% *w*/*v*, i.e., 5 kg of plant material in 100 L of water) or combined (2.5% *w*/*v* of each aqueous extract), was effective in lowering the numbers of the pest *Helicoverpa armigera* in a chickpea crop field, while not causing damage to the chickpea plants and increasing the grain yield. The authors suggest that farmers should spray their crops with the mentioned aqueous extracts twice with 15 days intervals between applications, starting on the 61st day after planting, for maximum results in controlling *Helicoverpa armigera* infestations. It can be highlighted that the extracts exhibit similar activity to the insecticide deltamethrin (25 g/L applied at recommended dose of 250 mL/ha) used as control (in the second application, the mean of larvae per plant before the treatment with combined aqueous extracts was 2.6 ± 0.80 and for the insecticide 2.8 ± 0.64, while 3 days after the treatment the means were of 0.73 ± 0.11 and 0.93 ± 0.23, respectively. In addition, the same study also found that the ethanolic extract of *Azadirachta indica* could be a viable pest control option since it was equally effective as the aqueous extracts or the insecticide (on the second application, the larvae/plant mean value before the treatment with ethanolic extract was 2.13 ± 0.41 decreasing to 0.86 ± 0.11 3 days after the treatment).

In a recent field study [74] aqueous extracts of five plants (*Capsicum annuum*, *Carica papaya*, *Lantana camara*, *Nicotiana tabacum* and *Tagetes minuta*) were investigated regarding their efficacy in controlling the population of *Aphis gossypii* present at an okra (*Abelmoschus esculentus* (L.) Moench) plantation over a four-month period. A visual scoring rating from 0 to 5 (where 0 meant no aphid presence and 5 meant presence of large continuous colonies) was used to determine the degree of aphid infestation. All plant extracts exerted better action against the pest than the reference insecticide mercaptothion. Better results were obtained at the second month, with *Carica papaya* presenting the best insecticide action with a score of 0.05, followed closely by *Tagetes minuta* (0.06). The remaining plants scored 0.09 (*Nicotiana tabacum*), 0.12 (*Lantana camara*) and 0.16 (*Capsicum annuum*), while the control insecticide scored 0.93. Despite the interesting and promising results obtained by the diverse plant extracts, the study is faulty due to the lack of robustness and reliability in the data presented, since the used system for determination of the degree of aphid infestation (scores from 0 to 5) is prone to the subjectivity of those who are collecting the data. A more rigours and precise approach (e.g*.,* counting the number of individuals) would have provided more assurance to extrapolate conclusions for comparison with other works.

### 2.2. Ethanolic Extracts

One of the few published works that uses the *Yponomeuta malinellus* plague as a target is the Ertürk and colleagues work [75]. In this study, an 8-day assay demonstrated the antifeedant activity exerted over 3–4^th^ instar larvae of *Yponomeuta malinellus* by ethanolic extracts derived from six different plants (flowers, leaves and stems combined) 1:5 ratio (50 g powder plant material: solvent): i.e., *Achillea coarctata* Poir., *Arum italicum* Mill., *Buxus sempervirens* L., *Liquidambar orientalis* Mill., *Tanacetum vulgare* L. and *Ocimum basilicum*. *Liquidambar orientalis* provided the highest antifeedant effect with a coefficient of deterrence of 80.90%, followed by *Tanacetum vulgare* (46.12%) and *Buxus sempervirens* (40.11%). Unfortunately, this work loses impact because it is lacking a reference insecticide as control to allow for better comparison with other published results and better evaluation of its potential as bio-insecticides.

The ethanolic extracts (0.05% *w*/*w*) of leaves and stems from four *Psychotria* species, i.e., *Psychotria capitata* Ruiz & Pav., *Psychotria goyazensis* Müll.Arg., *Psychotria hoffmannseggiana* (Willd. ex Schult.) Müll.Arg. and *Psychotria prunifolia* (Kunth) Steyerm., proved to be very effective against the pests *Sitophilus zeamais* and *Spodoptera frugiperda*. *Sitophilus zeamais* was very sensitive to the tested extracts, with a mortality rate of 100% after 3 days of contact with both leaves and stem extracts of *Psychotria prunifolia*, with leaves extract of *Psychotria capitata* and with stem extract of *Psychotria hoffmannseggiana*. Regarding *Spodoptera frugiperda*, the mortality caused by the leaves and stems extracts ranged from 80.00% to 95.83% at the end of an 11-day assay [76].

*Spodoptera frugiperda* was also the target species of a study aiming to assess the insecticide properties of the ethanolic extract of *Euphorbia pulcherrima* Willd. ex Klotzsch leaves [77]. Larvae of *Spodoptera frugiperda* were fed for 12 days an artificial liquid diet containing 0.5 or 1% (*w*/*v*) concentrations of the extract, resulting in impaired development of the larvae. Larval stage was increased by 19.657 ± 0.712 days with 0.5% concentration extract, while simultaneous reducing by 40% the larval weight in both cases. Larval mortality caused by 0.5% and 1% extracts was of, respectively, 24% and 26%. In addition, the 1% extract also affected *Spodoptera frugiperda* fertility, leading to a reduced number of the pest eggs. This study reveals a very active extract since it is one of the studies with the lowest applied concentration with significant effect, unfortunately it lacks the use of a reference insecticide, thus the true value of these extracts as bio-insecticides and the comparisons with other similar works are diminished.

The ethanolic extracts of *Tagetes erecta* flower and leaf also demonstrated to affect *Spodoptera frugiperda* development once incorporated on their diet (500 ppm) [78]. The larval weight was reduced after a 14-day diet period, with untreated larvae weighing on average 0.3140 ± 0.1161 g, while larvae with leaf extract diet weighed on average 0.0746 ± 0.0089 g. In addition, flower extracts were responsible for the highest rate of *Spodoptera frugiperda* mortality in the assay (i.e., 88%).

A different work also evaluated the insecticide potential of *Tagetes erecta* [79]. The ethanolic extract of its flowers was tested against the stored crop insect pest *Tribolium castaneum* at larvae and adult stages. The LC_50_ for the 1^st^ instar larvae was of 31.86 µg/cm^2^ at 72 h and for the adult stage was of 149.34 µg/cm^2^ at 72 h. The authors opted to present these units since they were calculated by measuring the dry weight of the extract present in 1 mL that was applied into the Petri dish divided by the surface area of the respective Petri dishes. This is slightly problematic because it does not allow comparison with different works.

*Azadirachta indica* ethanolic extract was the best of nine extracts whose effectiveness in protecting chickpea seeds against the pulse beetle *Callosobruchus chinensis* was evaluated [61]. This extract at 10% v/w concentration caused, after a seven days treatment, the decrease in the number of adult insects (25.67 ± 1.40), laid eggs per 100 seeds (15.67 ± 1.19) and infestation percentage (9.24%) when compared with the control (190 ± 2.28; 94.33 ± 1.97 and 63.57%, respectively).

An extensive work carried out by Chermenskaya et al. [80] analysed the insecticide and feeding deterrent potential of 139 ethanolic extracts (1% *w*/*v* concentration) of different morphological parts of 123 plants, applied over young bean leaves (*Phaseolus vulgaris*), against three agricultural pests: western flower thrips (*Frankliniella occidentalis*), grain aphid (*Shizaphis graminum*) and two-spotted spider mite (*Tetranychus urticae*). *Frankliniella occidentalis* proved to be highly tolerant to the plant extracts, with only 12 plants showing some activity. The extracts of the aerial parts of *Plantago major* L. and *Silene sussamyrica* Lazkov presented the best activities, causing reduction in the numbers of the species after five days of treatment by 41.9% and 50.2%, respectively. In contrast, the other pest species tested were way more susceptible to the plant extracts. The numbers of *Shizaphis graminum* were reduced by 69 plant extracts, with 8 of them showing high insecticidal activity (over 80% mortality). *Ungernia sewerzowii* (Regel) B. Fedtsch. (root extract), *Anabasis aphylla* L. (twigs extract) and *Ferula foetida* (Bunge) Regel. (root extract) caused a mortality of 89.1, 95.1 and 100%, respectively, 24 h after leaves treatment, and proved to be the best extracts against *Shizaphis graminum*. Regarding *Tetranychus urticae*, 75 plant extracts were effective in lowering their numbers over seven days of treatment with 7 plant extracts showing high insecticidal activity (over 80% mortality). The most active extracts were those of the roots of *Convolvulus krauseanus* Regel. and Schmalh. (95.6% mortality) and the leaves of *Ailanthus altissima* (Mill.) Swingle (97.4% mortality). In addition, the feeding deterrent results carried out with the extracts that presented efficacy over 80% showed that the *Ailanthus altissima*, *Allium obliquum* L. and *Vinca erecta* Regel & Schmalh. plant extracts were 100% effective in deterring the spider mites, while the *Anabasis aphylla* twig extract was the most effective (81.1%) in deterring the grain aphid. Extracts from seeds and twigs of *Anabasis aphylla*, roots of *Aconitum soongaricum* (Regel) Stapf and aerial parts of *Prangos lipskyi* Korovin proved to be the most effective, with over 90% of feeding deterring against western flower thrips. A flaw in this article is that, despite the numerous extracts tested that allow to obtain several valuable results, the authors could have standardized the presentation of results, e.g., in detailed tables, and not have the results spread over several tables, graphics and scattered along the article, which makes it difficult to understand the data obtained.

*Syzygium cumini* (L.) Skeels ethanolic extract at concentrations of 75, 150 and 300 µg/mL were also tested to control the population of *Tetranychus urticae*. The obtained mortality rates were 35.00 ± 0.31%, 85.00 ± 0.64% and 100.00 ± 0.00%, while the LC_50_ value was established at 98 µg/mL [81]. It should be noted that the insecticide effects observed in this study were obtained using much lower concentrations (about 100 times lower) than what is generally reported by other studies. It would have been extremely relevant to report a result obtained under the same experimental conditions using a pesticide of reference in order to validate this level of activity. Numa and colleagues [82] also used *Tetranychus urticae* as target species on the evaluation of the potential insecticidal effect of the ethanolic extract of *Cnidoscolus aconitifolius* (Mill.) I.M.Johnst. leaves. The mortality and oviposition of individuals were recorded over a 72 h period after exposure to the extracts at various concentrations (10 to 2000 µg/mL), being notorious an increased mortality and a reduced fertility in a dose-dependent manner. The LC_50_ of 24, 48 and 72 h were ascertained at 1223.637 ± 47.85 µg/mL, 990.37 ± 44.24 µg/mL and 901.25 ± 41.54 µg/mL, respectively.

Zhang et al. [83] conducted a study evaluating the insecticide potential of powdered tubers of an unusual plant (*Pinellia ternata* (Thunb.) Makino) against the beet armyworm *Spodoptera exigua*, a target also little used. They reported that the ethanol extract inhibited feeding from 43.07% to 86.78% at concentrations ranging between 12.5 and 100 mg/mL, with a LC_50_ of 43.594 mg/mL after 48 h of treatment. In addition, for the same concentration range, oviposition deterrent activity was between 39.69% and 78.34%.

In addition to aqueous extracts (see previous section), ethanolic extracts have also been tested to combat the *Plutella xylostella* pest. Silva and colleagues [84] evaluated the effect of various ethanolic extracts from leaves and stems mainly of *Croton* species (i.e*., Croton jacobinensis* Baill., *Mallotus rhamnifolius* (Willd.) Müll.Arg. (syn. *Croton rhamnifolius* Willd.) and *Croton sellowii* Baill.) against the diamondback moth (*Plutella xylostella*). All extracts proved to be toxic (LC_50_ values of 14.95 to 1252.00 µg/mL) with the leaf extract from *Mallotus rhamnifolius* demonstrating the greatest toxicity (LC_50_ = 14.95 µg/mL), followed by its stem extract (LC_50_ = 42.40 µg/mL). If the authors had added a control with a reference insecticide the study would have gain extra scientific power, but this was not the case.

Cabbage aphid *Brevicoryne brassicae* is a very common target in bio-pesticides studies. A recent work [85] evaluated the ethanolic leaf extracts (2.5 to 20 mg/mL) of *Artemisia argyi* H.Lév. & Vaniot, *Cannabis sativa* L. (syn. *Cannabis indica* Lam.) and *Citrullus colocynthis* (L.) Schrad. effects against the pest *Brevicoryne brassicae*. The toxicity results demonstrated that the *Artemisia argyi* extract was the most active (LC_50_ value of 3.91 mg/mL), followed by *Citrullus colocynthis* and *Cannabis sativa* with, respectively, LC_50_ values of 6.26 mg/mL and 10.04 mg/mL.

In addition to the most relevant studies discussed above, other studies involving other target pests and aqueous and ethanolic extracts from other plants are published and deserve to be mentioned in this review. Many of these studies involve simpler experimental designs, especially the activity shown by the plant under study and the target species used. In order to provide a more clear and direct reading, the following table (Table 1), alphabetically organized by the taxonomic name of the evaluated plant species, summarizes the most relevant information regarding the aqueous and ethanolic plant extracts with potential as new bio-insecticides. Two additional columns were added to clarify the plant part used and the extraction processes involved in each reference, providing sufficient information to allow for easy replication of the results obtained by anyone who seeks this kind of knowledge to overcome pest issues.

The above table (Table 1) addresses 95 plants whose aqueous and/or ethanolic extracts were reported in scientific studies as presenting some sort of insecticide property. Despite the effort to reconcile all the information in the best possible way, to facilitate its reading and to allow conclusions between the different studies, data comparisons are limited by the different and non-convertible units (e.g., plant mass per volume vs. extract mass per volume) that many authors chose to present their results and by the methodologies used in each work.

For instance, 14 different non-comparable categories (some of them with their unique units) could be used to express the insecticide property of an extract throughout the covered studies (i.e., Number of Leaves Damaged, Damage Index, Damage Rate, Life Expectancy, Survival Rate, Mortality Rate, LC_50_, Infestation Rate, Infestation Score, Insect Number Reduction Rate, Insect Number Reduction, Antifeedant and Deterrence Index, Weight Reduction and Larvae/Plant Ratio). Thus, comparison of results can only be made according to other studies from the same category of insecticide property, e.g., *Capsicum annuum* aqueous extract caused a reduction of 45.94% of *Hellula undalis* population [71] while *Ageratum conyzoides* caused a 100% reduction of *Plutella xylostella* population [66]. Furthermore, different species react in different ways, whereby conclusions should be made with caution regarding the potential of a particular extract from a given plant against a specific pest. Regardless of that, some conclusions can be made analysing Table 1 without impairments and will be discussed in the next section.

## 3. Final Considerations and Future Perspectives

The increasing global demand for food production results in an immense pressure to produce faster and bigger crop yields, leading to an excessive use of synthetic pesticides, from smallholders to big farms, without concerns for the long-term effects. In order to combat the negative consequences of synthetic pesticides use, without jeopardizing agricultural outcome, a search for effective alternatives began in the last decades and is currently ongoing. Plants produce remarkable compounds to overcome the challenges imposed by nature and are seen as the main source of answers to this problem.

Regarding plants used to protect crops in traditional agriculture, several examples can be given with various species being used in different ways, depending on the location. *Azadirachta indica* and *Nicotiana tabacum* can be highlighted as two of the plants reported regularly, being vastly rooted in agricultural popular knowledge. Therefore, it is not surprising that both plants belong to the most targeted plants for research in bio-insecticides, with interesting results consolidating their practices in traditional farming knowledge.

The literature analysis of studies that addressed the task of ascertaining the insecticide properties of aqueous and/or ethanolic plant extracts allowed for the identification of 95 plants with reported and validated insecticide activity, having potential to be viable options in the pest management approach, after further studies. From the 95 plants, four can be highlighted as the most researched ones (i.e., *Azadirachta indica*, *Capsicum annuum*, *Nicotiana tabacum*, and *Tagetes erecta*) with *Azadirachta indica* being present at 7 different studies with promising results (e.g*.,* aqueous seed extract could reduce the survival period of *Aphis gossypii* from a life expectancy of 17.4 days to 2.5 days; 3 days of treatment with aqueous seed extract resulted in a lower larvae/plant ratio of *Helicoverpa armigera*).

To determine the insecticide potential of each extract from each plant, a total of 34 insect species, known for being crop pests, were used in the various works. *Brevicoryne brassicae* was by far the most targeted species, being tested against the aqueous and/or ethanolic extracts of 23 different plants. Other commonly targeted pests were *Plutella xylostella*, *Spodoptera frugiperda*, *Callosobruchus chinensis*, *Tetranychus urticae* and *Agrotis ipsilon*, tested against 14, 13, 11, 11 and 9 distinct plants, respectively.

Maceration with dried material (usually leaves) plus water or ethanol was the main choice of most authors, with some of them providing detailed information regarding quantities of plant material and solvents used. Unfortunately, in other cases authors are too vague when addressing the extraction method, sticking to just stating “plant material soaked in solvent for 24 h” which is problematic for any reader trying to replicate procedures, compare results and deduce conclusions.

In addition, other plants not included here can also be targeted for future works since they also possess insecticide properties, but their aqueous and/or ethanolic extracts are not yet investigated, i.e., plants with insecticide activities exhibited by other organic extracts [21,91], essential oils [92,93] and pure compounds [45,94]. Other plants that should be considered for future research are the ones that proved their insecticide property through other techniques, e.g., powders [95], or against other insects that are not seen as agricultural pests, such as mosquitos [96,97], termites [98,99] and ticks [100,101]. In addition, plants know as companion plants should also be considered since they act many times as repellent for specific insects [20,102]. Furthermore, it should be noted the existence of published studies with interesting results and experimental designs using aqueous and ethanolic plant extracts but obtained through more complex extraction techniques (e.g*.,* soxhlet) that could also be taken into account in the search for new bio-insecticides but that fall out of the scope of this review [103,104,105,106,107].

This review shows that, in addition to the choice of the target plant and pest, most published works do not take into account an integrated vision of bio-insecticides uses. In fact, few are the studies that include variables in their experimental design such as phytotoxicity and effect on plant growth, impact on non-targeted species such as bees and effects on human health through their consumption [52,108]. The evaluation of long-term use of bio-insecticides in the surrounding place is imperative and some form of safety assessment needs to be considered as it could have serious repercussions. Studies carried out with plant extracts of organic solvents that have residual toxicity, such as methanol [109], are not useful in the field as alternatives to the existing environmentally hazardous synthetic pesticides, thus only works with aqueous and/or ethanolic plant extracts should be the norm. In addition, field studies where real farmers learn from real scientists the reasons to change their habits should be prioritized over laboratory ones [110], because despite good results in controlled laboratory conditions, when put into practice on the field by farmers themselves, results may be unpredictable, causing a waste of time and money and the return to the use of synthetic pesticides.

Regardless of the above suggestions to improve research in this subject and ensure better formulations of bio-insecticides, allowing for sufficient persistence of their stability, quality and insecticidal effect, there is still a major obstacle that only delays the adoption of bio-insecticides in the detriment of conventional synthetic insecticides. Probably the biggest impairment is the regulatory environments that changes according to countries legislations [111]. Health and environmental problems caused by insecticides in the past are the common denominators that lead to strict criteria for the registration of new insecticides, e.g., in Europe [112] and in the USA [113]. As defined by European Regulation (EC No. 1107/2009) the adoption of new bio-insecticides based on active substances (pure compounds with, in this case, insecticide properties) and basic substances (i.e*.,* other registered bio-products which may be useful for plant protection) should demonstrate and guarantee that they are not carcinogenic, corrosive or skin sensitisers, endocrine-disrupting, immunotoxic, mutagenic and not neurotoxic and they should not have any unacceptable effect on the environment [114]. These restrictions are important and should not be neglected, however, the bureaucracy involved must be revised to facilitate the process of adopting new bio-insecticides. Although there are already some bio-insecticides registered and authorized, being commercialized and used [115] they still represent a very small niche of the market [116] Faster and easier implementation of bio-insecticides research results is imperative, but regulatory requirements are so time consuming and costly that only large agrochemical companies have the resources to do so, thus, perpetuating the synthetic insecticides widespread agricultural use [21,117].

Quoting Isman in his most recent work [47]: “Perhaps it is time to refocus the attention of the research community toward the development and application of known botanicals rather than to screen more plants and isolate further novel bioactive substances that satisfy our curiosity but are unlikely to be of much utility”. This review, as stated in the introduction, contemplates only studies performed without complex and/or expensive apparatus, with easily accessible solvents, such as ethanol and/or water, which may later be reproduced by farmers themselves. This enables a bridge to be established between raw scientific data and a more practical reality.

Taking all the information stated above, the truth is that plants are an enormous source of secondary metabolites that can have tremendous impact in wide sectors of human society; it remains to be known where to look for answers, but hopefully this review can help with that.

## Figures and Tables

**Table 1:** Plant species mentioned in literature with potential to be effective bio-insecticides, according to their aqueous and/or ethanolic extracts pesticide properties.

Plant Species	Extract	Part of the Plant Used	Extract Preparation	Target Insect Species	Activity *	References
*Achillea coarctata* Poir.	Ethanolic	Flowers; Leaves; Stems	Maceration (50 g DW + EtOH at 1:5 ratio)	*Yponomeuta malinellus*	Deterrence index = 24.65%	[75]
*Aconitum soongaricum* (Regel) Stapf	Aqueous- ethanolic	Roots	Maceration (30 g DW + 300 mL EtOH), aqueous emulsions at a concentration of 1%	*Frankliniella occidentalis*	Deterrence index over 90%	[80]
*Ageratum conyzoides* (L.) L.	Aqueous	Leaves	Maceration (30 g FW + 1 L tap water)	*Brevicoryne brassicae;* *Plutella xylostella*	100 ± 0.00% *P. xylostella* number reduction (3% *w*/*v*)	[66]
*Ailanthus altissima* (Mill.) Swingle	Aqueous- ethanolic	Leaves	Maceration (30 g DW + 300 mL EtOH), aqueous emulsions at a concentration of 1%	*Tetranychus urticae*	97.4% *T. urticae* number reduction after 7 days of treatment	[80]
*Allium obliquum* L.	Aqueous- ethanolic	Whole plant	Maceration (30 g DW + 300 mL EtOH), aqueous emulsions at a concentration of 1%	*Tetranychus urticae*	100% deterrence index over *T. urticae*	[80]
*Allium sativum* L.	Aqueous	Bulb	Maceration (FW + H_2_O at 1:1 ratio) [64]; Maceration (100 g DW + 1 L H_2_O) [69]; Maceration (100 g FW + 0.5 L H_2_O), H_2_O added until 2 L [71]	*Acalymma vittatum* [69]; *Brevicoryne brassicae* [71]; *Hellula undalis* [71]; *Plutella xylostella* [71]; *Polyphagotarsonemus latus* [69]; *Raphidopalpa foveicollis* [69]; *Tribolium castaneum* [64]; *Trichoplusia ni* [71]	LC_50_ against *T. castaneum* at 48 h = 90.8 µL/L air [64]; Average treated crop leaves attacked = 4.11 ± 0.77 (Average of leaves attacked in untreated crop = 4.66 ± 0.33) [69]; *B. brassicae* reduction of 42.05% (Control insecticide emamectin benzoate at 2.5 mL/L = reduction of 70.83%) [71]	[64,69,71]
*Ammi majus* L.	Ethanolic	Seeds	Maceration (250 g DW + 500 mL EtOH for 72 h)	*Agrotis ipsilon*	Antifeedant index after 24 h feeding on 5% extract-treated leaves = 56.4%	[86]
*Anabasis aphylla* L.	Aqueous- ethanolic	Seeds; Twigs	Maceration (30 g DW + 300 mL EtOH), aqueous emulsions at a concentration of 1%	*Shizaphis graminum*	95.1% *S. graminum* number reduction after 24 h of treatment (twigs extract)	[80]
*Annona squamosa* L.	Aqueous; Ethanolic	Seeds	Maceration (10 g DW + 100 mL H_2_O or EtOH)	*Callosobruchus chinensis*	Aqueous: *C. chinensis* infestation percentage = 23.77% after 7 days of 10% extract treatment; Ethanol: *C. chinensis* infestation percentage = 19.55% after 7 days of 10% extract treatment (Control: *C. chinensis* infestation percentage = 63.57% after 7 days treatment)	[61]
*Anthemis cotula* L.	Aqueous	Leaves	Maceration (50 mg FW + 1000 mL H_2_O)	*Mythimna separata*	Mortality rate = 80.71% (Control: Dichlorvos 76 EC (0.076%) mortality rate = 89.34%.)	[70]
*Aphanamixis polystachya* (Wall.) R.Parker	Aqueous; Ethanolic	Seeds	Maceration (10 g DW + 100 mL H_2_O or EtOH)	*Callosobruchus chinensis*	Aqueous: *C. chinensis* infestation percentage = 21.55% after 7 days of 10% extract treatment; Ethanol: *C. chinensis* infestation percentage = 32.62% after 7 days of 10% extract treatment (Control: *C. chinensis* infestation percentage = 63.57% after 7 days of treatment)	[61]
*Apium graveolens* L.	Ethanolic	Seeds	Maceration (250 g DW + 500 mL EtOH for 72 h)	*Agrotis ipsilon*	Antifeedant index after 24 h feeding on 5% extract-treated leaves = 56.6%	[86]
*Artemisia annua* L.	Aqueous	Leaves	Maceration (50 mg FW + 1000 mL H_2_O)	*Mythimna separata*	Mortality rate = 84.59% (Control: Dichlorvos 76 EC (0.076%) mortality rate = 89.34%.)	[70]
*Artemisia argyi* H.Lév. & Vaniot	Ethanolic	Leaves	Maceration (100 g DW + 3 × 400 mL EtOH for 72 h each)	*Brevicoryne brassicae*	LC_50_ = 3.91 mg/mL	[85]
*Arum italicum* Mill.	Ethanolic	Flowers; Leaves; Stems	Maceration (50 g DW + EtOH at 1:5 ratio)	*Yponomeuta malinellus*	Deterrence index = 26.95%	[75]
*Atraphaxis toktogulicum* (Lazkov) T.M. Schust. & Reveal (syn. *Polygonum toktogulicum* Lazkov)	Aqueous- ethanolic	Root	Maceration (30 g DW + 300 mL EtOH), aqueous emulsions at a concentration of 1%	*Shizaphis graminum*	84.0% *S. graminum* number reduction after 24 h of treatment	[80]
*Azadirachta indica* A.Juss.	Aqueous [58,59,60,61,62,72,73]; Ethanolic [61,73]	Leaves [62]; Seeds [58,59,60,61,72,73]	Aqueous: Maceration (23.8 to 1410.0 mg DW + 100 mL H_2_O) [58]; Infusion (25 g FW + 250 mL H_2_O) [59]; Maceration (1 kg DW + 1.76 L H_2_O) [60]; Maceration (10 g DW + 100 mL H_2_O) [61]; Maceration (100 g DW + 1 L H_2_O + 0.1% soap) [62]; Maceration (250 g DW + 17 L H_2_O + 10 g soap) [72]; Maceration (5 kg DW + 100 L H_2_O) [73]; Ethanolic: Maceration (10 g DW + 100 mL EtOH) [61]; Maceration (100 g DW + 100 mL EtOH) [73]	*Aphis gossypii* [58,60]; *Aphis nerii* [59]; *Callosobruchus chinensis* [61]; *Helicoverpa armígera* [73]; *Sahlbergella singularis* [72]; *Spodoptera frugiperda* [62]	*A. gossypii* life expectancy reduced from 17.4 to 2.5 days at 14.1 mg/mL [58]; 27% higher mortality after 24 h when compared to the control (water-sprayed plants) [59]; *A. gossypii* numbers lowered by ≈ 80% after 72 h [60]; Aqueous: *C. chinensis* infestation percentage = 14.61% after 7 days of 10% extract treatment; Ethanolic: *C. chinensis* infestation percentage = 9.24% after 7 days of 10% extract treatment (Control: *C. chinensis* infestation percentage = 63.57% after 7 days of treatment) [61]; *S. frugiperda* larvae mortality over 50% at 10% extract [62]; *S. singularis* average number decreased after 5-month extract application at 14.7 g/L from 5.33 ± 0.33 to 1.21 ± 0.11 (Control: Actara^®^25 WG at 0.26 g/L from 4.83 ± 0.70 to 1.67 ± 0.13; untreated plants from 6.33 ± 1.14 to 8.95 ± 1.18) [72]; Aqueous: larvae/plant mean value before the treatment = 2.53 ± 0.64 and 3 days after the treatment = 0.93 ± 0.30; Ethanol: larvae/plant mean value before the treatment = 2.13 ± 0.41 and 3 days after the treatment = 0.86 ± 0.11 (Control insecticide deltamethrin 25 g/L applied at recommended dose of 250 mL/ha = 2.8 ± 0.64 before treatment and 0.93 ± 0.23 after 3 days) [73]	[58,59,60,61,62,72,73]
*Brugmansia suaveolens* (Humb. & Bonpl. ex Willd.) Bercht. & J.Presl	Aqueous	Flowers; Leaves	Infusion (FW + H_2_O) and Maceration (DW + H_2_O) both at 5% *w*/*v*	*Brevicoryne brassicae*	*B. brassicae* average survival at 10% concentration (leaves) = 2.1 days (Control = 6.2 days)	[55]
*Buxus sempervirens* L.	Ethanolic	Flowers; Leaves; Stems	Maceration (50 g DW + EtOH at 1:5 ratio)	*Yponomeuta malinellus*	Deterrence index = 40.11%	[75]
*Cannabis sativa* L. (syn. *Cannabis indica* Lam.)	Ethanolic	Leaves	Maceration (100 g DW + 3 × 400 mL EtOH for 72 h each)	*Brevicoryne brassicae*	LC_50_ = 10.04 mg/mL	[85]
*Capsicum annuum* L. [74] (syn. *Capsicum frutescens* L. [66,71])	Aqueous [66,71,74]; Aqueous- ethanolic [80]	Fruits [66,71,74]; Leaves [80]	Maceration (30 g FW + 1 L tap water) [66]; Maceration (140 g FW + 0.5 L H_2_O), H_2_O added until 2L [71]; Maceration (300 g FW + 20 L H_2_O) [74]; Maceration (30 g DW + 300 mL EtOH), aqueous emulsions at a concentration of 1% [80]	*Aphis gossypii* [74]; *Brevicoryne brassicae* [66,71]; *Hellula undalis* [71]; *Plutella xylostella* [71]; *Tetranychus urticae* [80]; *Trichoplusia ni* [71]	93 ± 0.06% *P. xylostella* number reduction (3% *w*/*v* concentration) [66]; *H. undalis* reduction of 45.94% (Control insecticide emamectin benzoate at 2.5 mL/L = reduction of 60.06%) [71]; *A. gossypii* infestation score at 2nd month = 0.16 (Control insecticide mercaptothion infestation score at 2nd month = 0.93) [74]; 85.6% *T. urticae* number reduction after 7 days of treatment [80]	[66,71,74,80]
*Carica papaya* L.	Aqueous	Leaves	Maceration (300 g DW + 20 L H_2_O)	*Aphis gossypii*	Infestation score at 2nd month = 0.05 (Control insecticide mercaptothion infestation score at 2nd month = 0.93)	[74]
*Cascabela thevetia* (L.) Lippold (syn. *Thevetia peruviana* (Pers.) K.Schum.)	Aqueous	Seeds	Maceration (250 g DW + 17 L H_2_O + 10 g soap)	*Sahlbergella singularis*	*S. singularis* average number decreased after 5-month extract application at 14.7 g/L from 5.33 ± 0.33 to 1.21 ± 0.11 (Control: Actara^®^25 WG at 0.26 g/L caused decrease from 4.83 ± 0.70 to 1.67 ± 0.13; untreated plants increased average number from 6.33 ± 1.14 to 8.95 ± 1.18)	[72]
*Catharanthus roseus* (L.) G.Don (syn. *Vinca rosea* L.)	Ethanolic	Aerial	Maceration (250 g DW + 500 mL EtOH for 72 h)	*Agrotis ipsilon*	Antifeedant index after 24 h feeding on 5% extract-treated leaves = 72.2%	[86]
*Cestrum elegans* (Brongn. ex Neumann) Schltdl.	Ethanolic	Aerial	Maceration (250 g DW + 500 mL EtOH for 72 h)	*Agrotis ipsilon*	Antifeedant index after 24 h feeding on 5% extract-treated leaves = 59.2%	[86]
*Chromolaena odorata* (L.) R.M.King & H.Rob.	Aqueous	Leaves	Maceration (30 g FW + 1 L tap water)	*Brevicoryne brassicae;* *Plutella xylostella*	100 ± 0.00% *P. xylostella* number reduction (3% *w*/*v* concentration)	[66]
*Citrullus colocynthis* (L.) Schrad.	Ethanolic	Leaves	Maceration (100 g DW + 3 × 400 mL EtOH for 72 h each)	*Brevicoryne brassicae*	LC_50_ = 6.26 mg/mL	[85]
*Cnidoscolus aconitifolius* (Mill.) I.M.Johnst.	Ethanolic	Leaves	Maceration (DW + EtOH)	*Tetranychus urticae*	72 h LC_50_ = 901.25 ± 41.54 µg/mL	[82]
*Convolvulus krauseanus* Regel. & Schmalh.	Aqueous- ethanolic	Roots	Maceration (30 g DW + 300 mL EtOH), aqueous emulsions at a concentration of 1%	*Tetranychus urticae*	95.6% *T. urticae* number reduction after 7 days of treatment	[80]
*Coriandrum sativum* L.	Aqueous	Leaves	Infusion (FW + H_2_O) and Maceration (DW + H_2_O)	*Brevicoryne brassicae;* *Myzus persicae*	*B. brassicae* and *M. persicae* survival rate at 10% concentration after 72 h = 0% (Control insecticide = 0%)	[56]
*Crotalaria juncea* L.	Aqueous; Ethanolic	Seeds	Maceration (10 g DW + 100 mL H_2_O or EtOH)	*Callosobruchus chinensis*	Aqueous: *C. chinensis* infestation percentage = 30.58% after 7 days of 10% extract treatment; Ethanol: *C. chinensis* infestation percentage = 25.77% after 7 days of 10% extract treatment (Control: *C. chinensis* infestation percentage = 63.57% after 7 days of treatment)	[61]
*Croton jacobinensis* Baill.	Ethanolic	Leaves; Stem	Maceration (DW + EtOH)	*Plutella xylostella*	LC_50_ stem extract = 116.21 µg/mL	[84]
*Croton sellowii* Baill.	Ethanolic	Leaves; Stem	Maceration (DW + EtOH)	*Plutella xylostella*	LC_50_ leaves extract = 80.1.36 µg/mL	[84]
*Cymbopogon citratus* (DC.) Stapf	Aqueous	Leaves	Maceration (100 g DW + 1 L H_2_O + 0.1% soap)	*Spodoptera frugiperda*	Larvae mortality over 50% at 10% extract	[62]
*Datura stramonium* L.	Aqueous	Leaves	Maceration (50 mg FW + 1000 mL H_2_O)	*Mythimna separata*	Mortality rate = 79.17% (Control: Dichlorvos 76 EC (0.076%) mortality rate = 89.34%.)	[70]
*Dodonaea viscosa* subsp. *angustifolia* (L.f.) J.G.West (syn. *Dodonaea angustifolia* L.f.)	Aqueous	Leaves	Maceration (1 kg + H_2_O)	*Earias vittella*	Damage crop dropped from 48.2% to 26.0% after 10% extract application (Control insecticide endosulfan = damage crop from 46.7% to 28.1%)	[87]
*Equisetum hyemale* L.	Aqueous	Leaves	Infusion (FW + H_2_O) and Maceration (DW + H_2_O)	*Brevicoryne brassicae*	*B. brassicae* survival rate at 10% concentration after 72 h = 1.5% (Control insecticide = 0%)	[56]
*Euphorbia pulcherrima* Willd. ex Klotzsch	Ethanolic	Leaves	Maceration (DW + EtOH) [77]; Maceration (250 g DW + 500 mL EtOH for 72 h) [86]	*Agrotis ipsilon* [86]; *Spodoptera frugiperda* [77]	12 days diet with 0.5% extract reduced larval weight by 40% [77]; Antifeedant index after 24 h feeding on 5% extract-treated leaves = 50.6% [86]	[77,86]
*Ferula foetida* (Bunge) Regel.	Aqueous- ethanolic	Root	Maceration (30 g DW + 300 mL EtOH), aqueous emulsions at a concentration of 1%	*Shizaphis graminum*	100% *S. graminum* number reduction after 24 h of treatment	[80]
*Jatropha curcas* L.	Aqueous [61,66]; Ethanolic [61]	Leaves [66]; Seeds [61]	Maceration (10 g DW + 100 mL H_2_O or EtOH) [61]; Maceration (30 g FW + 1 L tap water) [66]	*Brevicoryne brassicae* [66]; *Callosobruchus chinensis* [61]; *Plutella xylostella* [66]	Aqueous: *C. chinensis* infestation percentage = 35.87% after 7 days of 10% extract treatment; Ethanol: *C. chinensis* infestation percentage = 43.37% after 7 days of 10% extract treatment (Control: *C. chinensis* infestation percentage = 63.57% after 7 days of treatment) [61]; *B. brassicae* infestation score = 0.00 ± 0.00 (3% *w*/*v* concentration) [66];	[61,66]
*Juglans regia* L.	Aqueous	Leaves	Maceration (50 mg FW + 1000 mL H_2_O)	*Mythimna separata*	Mortality rate = 69.82% (Control: Dichlorvos 76 EC (0.076%) mortality rate = 89.34%.)	[70]
*Lantana camara* L.	Aqueous	Leaves	Maceration (300 g DW + 20 L H_2_O)	*Aphis gossypii*	Infestation score at 2^nd^ month = 0.12 (Control insecticide mercaptothion infestation score at 2^nd^ month = 0.93)	[74]
*Limonium tianschanicum* Lincz.	Aqueous- ethanolic	Aerial	Maceration (30 g DW + 300 mL EtOH), aqueous emulsions at a concentration of 1%	*Shizaphis graminum*	82.2% *S. graminum* number reduction after 24 h of treatment	[80]
*Linum usitatissimum* L.	Aqueous	Seeds	Maceration (10 g DW + 100 mL H_2_O)	*Callosobruchus chinensis*	*C. chinensis* infestation percentage = 48.09% after 7 days of 10% extract treatment (Control: *C. chinensis* infestation percentage = 63.57% after 7 days of treatment)	[61]
*Lippia javanica* (Burm.f.) Spreng.	Aqueous	Leaves	Maceration (DW + H_2_O; 1% and 10% *w*/*v*) [54]; Maceration (100 g DW + 1 L H_2_O + 0.1% soap) [62]	*Aphis fabae* [54]; *Epicauta albovittata* [54]; *Epicauta limbatipennis* [54]; *Ootheca mutabilis* [54]; *Ootheca bennigseni* [54]; *Spodoptera frugiperda* [62]	Index of plant damage by *O. mutabilis* and *Ootheca bennigseni* of 0.30 (Controls: pesticide = 0.39 and untreated plants = 2.01) [54]; *S. frugiperda* larvae mortality over 50% at 10% extract [62]	[54,62]
*Liquidambar orientalis* Mill.	Ethanolic	Flowers; Leaves; Stems	Maceration (50 g DW + EtOH at 1:5 ratio)	*Yponomeuta malinellus*	Deterrence index = 80.90%	[75]
*Luffa cylindrica* (L.) M.Roem.	Ethanolic	Aerial	Maceration (250 g DW + 500 mL EtOH for 72 h)	*Agrotis ipsilon*	Antifeedant index after 24 h feeding on 5% extract-treated leaves = 68.2%	[86]
*Mallotus rhamnifolius* (Willd.) Müll.Arg. (syn. *Croton rhamnifolius* Willd.)	Ethanolic	Leaves; Stem	Maceration (DW + EtOH)	*Plutella xylostella*	LC_50_ leaves extract = 14.95 µg/mL	[84]
*Melia azedarach* L.	Aqueous [67]; Ethanolic [86]	Leaves [86]; Seeds [67]	Maceration (5 g DW + 100 mL tap water) [67]; Maceration (250 g DW + 500 mL EtOH for 72 h) [86]	*Agrotis ipsilon* [86]; *Brevicoryne brassicae* [67];	86.5% *B. brassicae* number reduction (5% *w*/*v* concentration) [67]; Antifeedant index after 24 h feeding on 5% extract-treated leaves = 80.3% [86]	[67,86]
*Mentha arvensis* L	Aqueous- ethanolic	Leaves	Maceration (30 g DW + 300 mL EtOH), aqueous emulsions at a concentration of 1%	*Tetranychus urticae*	86.9% *T. urticae* number reduction after 7 days of treatment	[80]
*Millettia ferruginea* (Hochst.) Baker	Aqueous	Seeds	Maceration (5 kg DW + 100 L H_2_O)	*Helicoverpa armigera*	Larvae/plant mean value before the treatment = 2.33 ± 1.10 and 3 days after the treatment = 0.80 ± 0.34 (Control insecticide deltamethrin 25 g/L applied at recommended dose of 250 mL/ha = 2.8 ± 0.64 before treatment and 0.93 ± 0.23 after 3 days)	[73]
*Nicotiana megalosiphon* Van Heurck & Müll.Arg.	Aqueous	Leaves	Maceration (5 g, 25 g and 50 g FW + 500 mL tap water)	*Brevicoryne brassicae*; *Myzus persicae*; *Plutella xylostella*	*P. xylostella* mortality rate of 100 ± 0.00% at 10% concentration after 24 h (Control insecticide tau-fluvalinate at 9.5 mL/L presented mortality rate of 22 ± 0.05%)	[57]
*Nicotiana tabacum* L.	Aqueous [56,61,62,66,74]; Ethanolic [61]	Leaves	Infusion (FW + H_2_O) and Maceration (DW + H_2_O) [56]; Maceration (10 g DW + 100 mL H_2_O or EtOH) [61]; Maceration (100 g DW + 1 L H_2_O + 0.1% soap) [62]; Maceration (30 g FW + 1 L tap water) [66]; Maceration (300 g DW + 20 L H_2_O) [74]	*Aphis gossypii* [74]; *Brevicoryne brassicae* [56,66]; *Callosobruchus chinensis* [61]; *Myzus persicae* [56]; *Plutella xylostella* [66]; *Spodoptera frugiperda* [62]	*B. brassicae* and *M. persicae* survival rate at 10% after 72 h = 0% (Control insecticide = 0%) [56]; Aqueous: *C. chinensis* infestation percentage = 24.51% after 7 days of 10% extract treatment; Ethanol: *C. chinensis* infestation percentage = 15.80% after 7 days of 10% extract treatment (Control: *C. chinensis* infestation percentage = 63.57% after 7 days of treatment) [61]; *S. frugiperda* larvae mortality over 50% at 10% extract [62]; *B. brassicae* infestation score = 0.00 ± 0.00 (3% *w*/*v*) [66]; *A. gossypii* infestation score at 2^nd^ month = 0.06 (Control insecticide mercaptothion infestation score at 2^nd^ month = 0.93) [74]	[56,61,62,66,74]
*Nicotiana tabacum* var. *virginica* (C. Agardh) Comes	Aqueous	Flowers; Leaves	Infusion (FW + H_2_O) and Maceration (DW + H_2_O) both at 5% *w*/*v*	*Brevicoryne brassicae*	*B. brassicae* average survival at 10% concentration (leaves) = 1.9 days (Control = 6.2 days)	[55]
*Ocimum basilicum* L.	Ethanolic	Flowers; Leaves; Stems	Maceration (50 g DW + EtOH at 1:5 ratio)	*Yponomeuta malinellus*	Deterrence index = 32.93%	[75]
*Ocimum gratissimum* L.	Aqueous [56,66]; Aqueous- ethanolic [88]	Leaves	Infusion (FW + H_2_O) and Maceration (DW + H_2_O) [56]; Maceration (30 g FW + 1 L tap water) [66]; Maceration (100 g DW + 300 mL EtOH), dissolved in H_2_O to prepare solutions of 1% to 4% concentration *w*/*v* [88]	*Acanthoscelides obtectus* [88]; *Brevicoryne brassicae* [56,66]; *Plutella xylostella* [66]	*B. brassicae* survival rate at 10% concentration after 72 h = 0.8% (Control insecticide = 0%) [56]; 89 ± 0.05% *P. xylostella* number reduction (3% *w*/*v* concentration) [66]; *A. obtectus* mortality rate (4% extract) = 28.80% [88]	[56,66,88]
*Origanum vulgare* L.	Aqueous- ethanolic	Aerial	Maceration (30 g DW + 300 mL EtOH), aqueous emulsions at a concentration of 1%	*Shizaphis graminum*	80.9% *S. graminum* number reduction after 24 h of treatment	[80]
*Papaver rhoeas* L.	Aqueous- ethanolic	Aerial	Maceration (30 g DW + 300 mL EtOH), aqueous emulsions at a concentration of 1%	*Tetranychus urticae*	89.7% *T. urticae* number reduction after 7 days of treatment	[80]
*Persicaria hydropiper* (L.) Delarbre (syn. *Polygonum hydropiper* L.)	Aqueous; Ethanolic	Seeds	Maceration (10 g DW + 100 mL H_2_O or EtOH)	*Callosobruchus chinensis*	Aqueous: *C. chinensis* infestation percentage = 23.86% after 7 days of 10% extract treatment; Ethanol: *C. chinensis* infestation percentage = 37.62% after 7 days of 10% extract treatment (Control: *C. chinensis* infestation percentage = 63.57% after 7 days of treatment)	[61]
*Peumus boldus* Molina	Aqueous	Leaves	Maceration (10 g DW + 100 mL H_2_O)	*Helicoverpa zea*; *Spodoptera frugiperda*	*S. frugiperda* after 7 days of diet with 8.0% extract presented LC_50_ of 2.31 mL/kg	[63]
*Pinellia ternata* (Thunb.) Makino	Ethanolic	Tubers	Maceration (50 g DW + 1 L EtOH)	*Spodoptera exigua*	LC_50_ at 48 h of 43.594 mg/mL	[83]
*Pistacia vera* L.	Ethanolic	Leaves	Maceration (250 g DW + 500 mL EtOH for 72 h)	*Agrotis ipsilon*	Antifeedant index after 24 h feeding on 5% extract-treated leaves = 65.0%	[86]
*Plantago major* L.	Aqueous- ethanolic	Aerial	Maceration (30 g DW + 300 mL EtOH), aqueous emulsions at a concentration of 1%	*Frankliniella occidentalis*	41.9% *F. occidentalis* number reduction after 5 days of treatment	[80]
*Prangos lipskyi* Korovin	Aqueous- ethanolic	Aerial	Maceration (30 g DW + 300 mL EtOH), aqueous emulsions at a concentration of 1%	*Tetranychus urticae*	86.1% *T. urticae* number reduction after 7 days of treatment	[80]
*Psychotria capitata* Ruiz & Pav.	Ethanolic	Leaves; Stems	Maceration (FW + EtOH)	*Sitophilus zeamais*; *Spodoptera frugiperda*	Mortality rate of *S. zeamais* after 3 days (0.05%) = 100% (leaves extract)	[76]
*Psychotria goyazensis* Müll.Arg.	Ethanolic	Leaves; Stems	Maceration (FW + EtOH)	*Sitophilus zeamais*; *Spodoptera frugiperda*	Mortality rate of *S. frugiperda* after 11 (0.05%) days = 95.83% (stem extract)	[76]
*Psychotria hoffmannseggiana* (Willd. ex Schult.) Müll.Arg.	Ethanolic	Leaves; Stems	Maceration (FW + EtOH)	*Sitophilus zeamais*; *Spodoptera frugiperda*	Mortality rate of *S. zeamais* after 3 days (0.05%) = 100% (stem extract)	[76]
*Psychotria prunifolia* (Kunth) Steyerm.	Ethanolic	Leaves; Stems	Maceration (FW + EtOH)	*Sitophilus zeamais*; *Spodoptera frugiperda*	Mortality rate of *S. zeamais* after 3 days (0.05%) = 100% (both extracts)	[76]
*Ricinus communis* L.	Aqueous [61,66]; Ethanolic [61]	Leaves [66]; Seeds [61]	Maceration (10 g DW + 100 mL H_2_O or EtOH) [61]; Maceration (30 g FW + 1 L tap water) [66]	*Brevicoryne brassicae* [66]; *Callosobruchus chinensis* [61]; *Plutella xylostella* [66]	Aqueous: *C. chinensis* infestation percentage = 42.93% after 7 days of 10% extract treatment; Ethanol: *C. chinensis* infestation percentage = 42.38% after 7 days of 10% extract treatment (Control: *C. chinensis* infestation percentage = 63.57% after 7 days of treatment) [61]; *B. brassicae* infestation score = 0.00 ± 0.00 (3% *w*/*v* concentration) [66]	[61,66]
*Sapindus saponaria* L.	Aqueous	Seeds	Maceration (DW + H_2_O)	*Spodoptera frugiperda*	Larvae mortality of 63.15% after 14 days of feeding of treated corn leaves (1% *w*/*v*)	[89]
*Saponaria officinalis* L.	Aqueous	Roots	Maceration (100, 80, 60, 30 or 15 g DW + 1 L tap water)	*Tetranychus urticae*	LC_50_ for eggs = 0.31% *w*/*v*	[65]
*Senna sophera* (L.) Roxb. (syn. *Cassia sophera* L.)	Aqueous	Leaves	Maceration (30 g FW + 1 L tap water) [66]; Infusion and Maceration (100 g DW + 800 mL H_2_O) [68]	*Brevicoryne brassicae* [66]; *Callosobruchus maculatus* [68]; *Plutella xylostella* [66]	*B. brassicae* infestation score = 0.00 ± 0.00 (3% *w*/*v* concentration) [66]; Number of *C. maculatus* statistically significant reduced when compared with the control (*p* < 0.05) [68]	[66,68]
*Sida acuta* Burm.f.	Aqueous- ethanolic	Leaves	Maceration (100 g DW + 300 mL EtOH), dissolved in H_2_O to prepare solutions of 1% to 4% concentration *w*/*v*	*Acanthoscelides obtectus*	Mortality rate (4% extract) = 31.47%	[88]
*Silene sussamyrica* Lazkov	Aqueous- ethanolic	Aerial	Maceration (30 g DW + 300 mL EtOH), aqueous emulsions at a concentration of 1%	*Frankliniella occidentalis*	50.2% *F. occidentalis* number reduction after 5 days of treatment	[80]
*Solanum aculeatissimum* Jacq.	Aqueous	Fruits; Leaves	Infusion (FW + H_2_O) and Maceration (DW + H_2_O) both at 5% *w*/*v*	*Brevicoryne brassicae*	*B. brassicae* average survival at 1% concentration (fruits) = 3.3 days (Control = 6.2 days)	[55]
*Solanum bonariense* L. (syn. *Solanum fastigiatum* var. *fastigiatum*)	Aqueous	Flowers; Fruits; Leaves	Infusion (FW + H_2_O) and Maceration (DW + H_2_O) both at 5% *w*/*v*	*Brevicoryne brassicae*	*B. brassicae* average survival at 10% concentration (fruits) = 1.4 days (Control = 6.2 days)	[55]
*Solanum guaraniticum* A. St.-Hil. (syn. *Solanum fastigiatum* var. *acicularium* Dunal)	Aqueous	Flowers; Fruits; Leaves	Infusion (FW + H_2_O) and Maceration (DW + H_2_O) both at 5% *w*/*v*	*Brevicoryne brassicae*	*B. brassicae* average survival at 10% concentration (fruits) = 1.7 days (Control = 6.2 days)	[55]
*Solanum pseudocapsicum* L. (syn. *Solanum diflorum* Vell.)	Aqueous	Fruits; Leaves	Infusion (FW + H_2_O) and Maceration (DW + H_2_O) both at 5% *w*/*v*	*Brevicoryne brassicae*	*B. brassicae* average survival at 10% concentration (fruits) = 2.4 days (Control = 6.2 days)	[55]
*Swertia chirata* Buch.-Ham. ex Wall.	Aqueous	Leaves	Maceration (100 g DW + 1 L H_2_O)	*Polyphagotarsonemus latus*	Average treated crop leaves attacked = 1.67 ± 0.18 (Average of leaves attacked in untreated crop = 4.66 ± 0.33	[69]
*Swietenia mahagoni* (L.) Jacq.	Aqueous [61,69]; Ethanolic [61]	Seeds	Maceration (10 g DW + 100 mL H_2_O or EtOH) [61]; Maceration (100 g DW + 1 L H_2_O) [69]	*Callosobruchus chinensis* [61]; *Epilachna varivestis* [69]; *Polyphagotarsonemus latus* [69]	Aqueous: *C. chinensis* infestation percentage = 30.32% after 7 days of 10% extract treatment; Ethanol: *C. chinensis* infestation percentage = 37.40% after 7 days of 10% extract treatment (Control: *C. chinensis* infestation percentage = 63.57% after 7 days of treatment) [61]; Average treated crop leaves attacked = 2.22 ± 0.48 (Average of leaves attacked in untreated crop = 4.66 ± 0.33 [69]	[61,69]
*Synedrella nodiflora* (L.) Gaertn.	Aqueous	Leaves	Maceration (30 g FW + 1 L tap water)	*Brevicoryne brassicae;* *Plutella xylostella*	*B. brassicae* infestation score = 0.00 ± 0.00 (3% *w*/*v* concentration)	[66]
*Syzygium cumini* (L.) Skeels	Ethanolic	Fruits	Maceration (50 g FW + EtOH)	*Tetranychus urticae*	LC_50_ = 98 µg/mL	[81]
*Tagetes erecta* L.	Aqueous [61]; Aqueous- Ethanolic [90]; Ethanolic [61,78,79,90]	Flowers [78,79]; Leaves [61,78]; Whole plant [90]	Maceration (10 g DW + 100 mL H_2_O or EtOH) [61]; Maceration (500 g DW + EtOH)[78]; Maceration (1 kg DW + 5 L EtOH) [79]; Maceration (DW + absolute EtOH or EtOH / H_2_O (70/30) at a ratio *w*/*v* of 1:10) [90]	*Callosobruchus chinensis* [61]; *Sitophilus zeamais* [90]; *Spodoptera frugiperda* [78]; *Tribolium castaneum* [79]	Aqueous: *C. chinensis* infestation percentage = 41.90% after 7 days of 10% extract treatment; Ethanol: *C. chinensis* infestation percentage = 36.98% after 7 days of 10% extract treatment (Control: *C. chinensis* infestation percentage = 63.57% after 7 days of treatment) [61]; *S. frugiperda* mortality rate = 88% (leaf’s extract) [78]; At 72 h after treatment LC_50_ of *T. castaneum* larvae = 31.86 µg/cm^2^ [79]; Aqueous-ethanolic LC_50_ against *S. zeamais* = 12.59 mg/mL; Ethanolic LC_50_ against *S. zeamais* = 11.23 mg/mL [90]	[61,78,79,90]
*Tagetes minuta* L.	Aqueous	Leaves	Maceration (300 g DW + 20 L H_2_O)	*Aphis gossypii*	Infestation score at 2^nd^ month = 0.06 (Control insecticide mercaptothion infestation score at 2^nd^ month = 0.93)	[74]
*Talisia esculenta* (A. St.-Hil.) Radlk.	Aqueous	Seeds	Maceration (DW + H_2_O)	*Spodoptera frugiperda*	Larvae mortality of 26.71% after 14 days of feeding of treated corn leaves (1% concentration)	[89]
*Tanacetum cinerariifolium* (Trevir.) Sch.Bip. (syn. *Pyrethrum cinerariifolium* Trevir.)	Aqueous- ethanolic	Aerial	Maceration (30 g DW + 300 mL EtOH), aqueous emulsions at a concentration of 1%	*Shizaphis graminum*	80.2% *S. graminum* number reduction after 24 h of treatment	[80]
*Tanacetum vulgare* L.	Ethanolic	Flowers; Leaves; Stems	Maceration (50 g DW + EtOH at 1:5 ratio)	*Yponomeuta malinellus*	Deterrence index = 46.12%	[75]
*Telfairia occidentalis* Hook.f.	Aqueous- ethanolic	Leaves	Maceration (100 g DW + 300 mL EtOH), dissolved in H_2_O to prepare solutions of 1% to 4% concentration *w*/*v*	*Acanthoscelides obtectus*	Mortality rate (4% extract) = 15.20%	[88]
*Tephrosia vogelii* Hook.f.	Aqueous	Leaves	Maceration (DW + H_2_O; 1% and 10% *w*/*v*)	*Aphis fabae*; *Epicauta albovittata*; *Epicauta limbatipennis*; *Ootheca mutabilis*; *Ootheca bennigseni*	Index of plant damage by *O. mutabilis* and *Ootheca bennigseni* of 0.94 (Controls: pesticide = 0.39 and untreated plants = 2.01)	[54]
*Tithonia diversifolia* (Hemsl.) A.Gray	Aqueous	Leaves	Maceration (DW + H_2_O; 1% and 10% *w*/*v*)	*Aphis fabae*; *Epicauta albovittata*; *Epicauta limbatipennis*; *Ootheca mutabilis*; *Ootheca bennigseni*	Index of plant damage by *O. mutabilis* and *Ootheca bennigseni* of 1.09 (Controls: pesticide = 0.39 and untreated plants = 2.01)	[54]
*Ungernia sewerzowii* (Regel) B. Fedtsch.	Aqueous- ethanolic	Root	Maceration (30 g DW + 300 mL EtOH), aqueous emulsions at a concentration of 1%	*Shizaphis graminum*	89.1% *S. graminum* number reduction after 24 h of treatment	[80]
*Vernonia amygdalina* Delile	Aqueous [54]; Aqueous- Ethanolic [88]	Leaves	Maceration (DW + H_2_O; 1% and 10% *w*/*v*) [54]; Maceration (100 g DW + 300 mL EtOH), dissolved in H_2_O to prepare solutions of 1% to 4% concentration *w*/*v* [88]	*Acanthoscelides obtectus* [88]; *Aphis fabae* [54]; *Epicauta albovittata* [54]; *Epicauta limbatipennis* [54]; *Ootheca mutabilis* [54]; *Ootheca bennigseni* [54]	Average insect abundance of *O. mutabilis* and *Ootheca bennigseni* of 0.63 (Controls: pesticide = 0.37 and untreated plants = 2.45) [54]; *A. obtectus* mortality rate (4% extract) = 33.60% [88]	[54,88]
*Vinca erecta* Regel & Schmalh.	Aqueous- ethanolic	Aerial	Maceration (30 g DW + 300 mL EtOH), aqueous emulsions at a concentration of 1%	*Tetranychus urticae*	100% deterrence index over *T. urticae*	[80]
*Withania somnifera* (L.) Dunal	Ethanolic	Aerial	Maceration (250 g DW + 500 mL EtOH for 72 h)	*Agrotis ipsilon*	Antifeedant index after 24 h feeding on 5% extract-treated leaves = 63.0%	[86]
*Zingiber officinale* Roscoe	Aqueous	Rhizomes	Maceration (50 mg FW + 1000 mL H_2_O)	*Mythimna separata*	Mortality rate = 75.64% (Control: Dichlorvos 76 EC (0.076%) mortality rate = 89.34%.)	[70]

DW—Dry weight; EtOH—Ethanol; FW—Fresh weight; H_2_O—Distilled water. * Only the highest level of activity per plant per reference presented.
